# A Comparison Between Transcutaneous Bilirubin and Total Serum Bilirubin Levels for the Management of Jaundice in Preterm Neonates by Bland-Altman Plot

**DOI:** 10.7759/cureus.18442

**Published:** 2021-10-02

**Authors:** Santosh K Panda, Abhinav Gaurav, Palash Das, Natabar Swain, Soumini Rath

**Affiliations:** 1 Neonatology, Kalinga Institute of Medical Sciences, Bhubaneswar, IND; 2 Pediatrics, Kalinga Institute of Medical Sciences, Bhubaneswar, IND; 3 Pediatric Medicine, Kalinga Institute of Medical Sciences, Bhubaneswar, IND

**Keywords:** neonatal hyperbilirubinemia, transcutaneous bilirubin, jaundice, neonates, preterm

## Abstract

Objective

To compare the bilirubin levels measured by transcutaneous bilirubinometer and serum samples for the management of jaundice in preterm neonates.

Methods

The study was a prospective comparative observational study conducted in a tertiary care neonatal unit of Odisha from January 2019 to June 2020. All inborn and outborn neonates with a gestational age of 28^0/7^ weeks to 36^6/7^ weeks with the clinical diagnosis of neonatal jaundice were included in the study. Transcutaneous bilirubin (TcB) was estimated by Dragger jaundice meter JM-105 and simultaneously venous blood and total serum bilirubin levels (TSB) were measured by diazonium method. The comparison between TcB and TSB values was analyzed by direct linear correlation in scatter plot and Bland-Altman plot.

Results

A total of 167 preterm neonates (66, 28-33^6/7 ^and 111, 34-36^6/7^), with a mean gestational age 33.55 ±2.36 weeks and a mean birth weight of 1960 ± 613 grams, were analyzed. The mean TSB and TcB levels were 12.99 ± 3.47 mg/dl (min-max 4.9-18.3 mg/dl) and 14.156 ± 4.71 mg/dl (min-max 4-20.1 mg/dL), respectively. The TcB is excellently correlated with TSB with a correlation coefficient of r =0.948, p ≤0.001. The bias difference between TcB and TSB is -1.16 (95% CI: 2.35, -4.6) mg/dl. The correlation coefficients between 28-33^6/7^ weeks gestational age groups (r = 0.944) and 34-36^6/7^gestational age (r = 0.950) were similar.

Conclusion

TcB is well correlated with TSB level in preterm neonates. Hence, TcB can be used for the guidance of management in these neonates.

## Introduction

Neonatal hyperbilirubinemia is a common problem with almost 50% term and 80% of preterm neonates suffering from some amount of neonatal hyperbilirubinemia [1]. Recent reports have shown the visual assessment of jaundice to be unreliable and unsafe [2]. Total serum bilirubin (TSB) level is the gold standard for the diagnosis of neonatal hyperbilirubinemia and is vital for the effective treatment of neonatal jaundice. Frequent clinical monitoring and timely serum bilirubin testing are the best strategies for the prevention of bilirubin-induced neurological damage. The TSB estimation procedure involves invasive extraction of venous blood. In premature neonates, it is especially bothering as repeated phlebotomy causes iatrogenic anemia and also increases parental anxiety [3]. In recent years, transcutaneous bilirubinometry is being used for serum bilirubin screening of neonates >35 weeks after 24 hours of age. The first clinically appropriate and portable transcutaneous bilirubinometer was introduced by Yamanouchi and associates working with the Minolta Camera Company [4]. In recent years, the availability of newer equipment with advanced technology is used for the management of preterm jaundice. This study was carried out to measure transcutaneous bilirubin (TcB) levels in preterm jaundiced neonates between 28 and 37 weeks of gestational age and compare their values with TSB levels.

## Materials and methods

This study was a prospective comparative study carried out at a tertiary care neonatal center of Odisha between January 2019 and June 2020 after institutional ethical clearance. All inborn and outborn neonates with a gestational age of 28^0-7^ weeks to 36^6-7^ weeks with the clinical diagnosis of neonatal jaundice, requiring laboratory investigation for serum bilirubin, were included in the study, after taking informed consent from the parents. Neonates developing jaundice within the first 24 hours of life, and neonates who already received phototherapy treatment for neonatal jaundice and had raised direct serum bilirubin >2 mg/dl from blood investigation were excluded from the study. Neonates were screened by Dragger jaundice meter JM-105 and venous blood was obtained for TSB. The TSB is measured by the diazonium method. A total of three readings of the bilirubin meter were taken over the mid sternum area and the average of three readings was recorded in "mg/dl." For the entire study period, we ensured that the instrumentations, the methodology, and the calibrators are not changed in the laboratory. A single observation of both TcB and TSB of each participant neonate was analyzed in this study. The demographic parameters gestational age, birth weight, gender, mode of delivery, neonates blood group, maternal blood group, and G6PD status were collected from the neonatal case records. Neonates were followed every 12 hours by neonatologist and jaundice was managed as per National Institute for Health and Care Excellence guidelines.

Statistical analysis

All the continuous data were presented as mean ± standard deviation (SD) and categorical data as frequency and percentage. Pearson's correlation coefficient calculation and linear regression analysis were used to check the relationship between TcB and TSB. Bland-Altman plot was placed for qualitative assessment of variables. All the data analyses were performed using statistical software IBM SPSS version 20.0 (IBM Corp, Armonk, NY).

## Results

Observations and results

A total of 167 preterm neonates were enrolled with a mean gestational age of 33.55 ± 2.36 weeks and a mean birth weight of 1960 ± 613 grams. In this study, 69 (41.3%) were females, 66 (39.5%) were early preterm <34 weeks, and 44 (26.3%) were very-low-birth-weight neonates (birth weight < 1500 grams). Hyperbilirubinemia was related to blood group incompatibility in 27 (16.2%), 21 (12.6%) cases were G6PD deficiency, and 119 (71.25%) were idiopathic neonatal jaundice. The mean TSB and TcB levels were 12.99 ± 3.47 mg/dl (min-max 4.9-18.3 mg/dl) and 14.156 ± 4.71 mg/dl (min-max 4-20.1 mg/dl), respectively.

**Figure 1:**
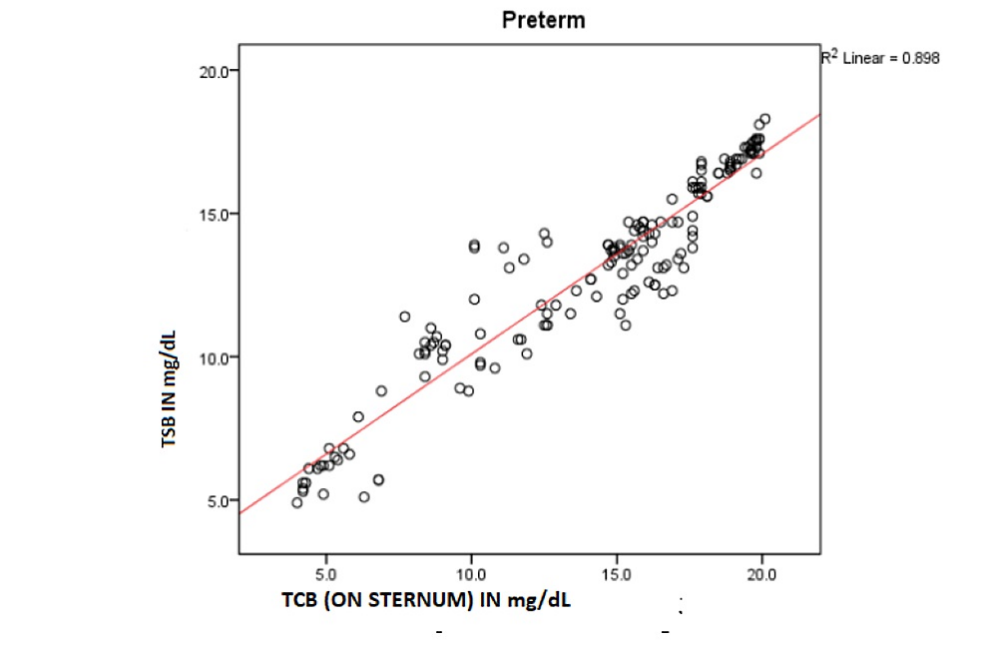
Scatter plot of TSB versus TcB (n = 167) TSB, total serum bilirubin; TcB, transcutaneous bilirubin.

The scatter plot of TSB versus TcB (Figure 1) shows a strong direct linear correlation. Pearson’s correlation coefficient, r = 0.948 and R^2^ = 0.898 with p ≤0.001. Simple linear regression is calculated as TSB = 3.121 + 0.698 × TcB. This equation can be used for predicting TSB from TcB with a high degree of accuracy.

**Figure 2:**
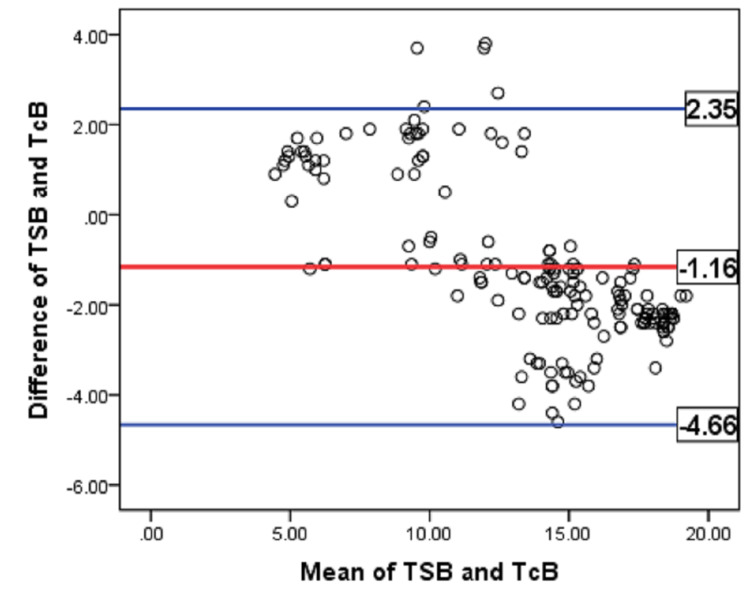
Bland-Altman plot of TSB versus TcB (n = 167) TSB, total serum bilirubin; TcB, transcutaneous bilirubin.

Agreement between TSB and TcB values is assessed by constructing Bland-Altman plot (Figure 2). X-axis suggests the mean of TSB and TcB. Y-axis was plotted by the difference between TSB and TcB. The bias line indicates a difference of 1.16 mg/dl between the averages of the two variables. Analysis of mean bilirubin level showed that TSB was 1.16 mg/dl lower than the measured TcB. The majority of the data points fall within ±1.96 times the SD of the difference between the TSB and TcB values. This corroborates that there is a strong agreement between the two variables.

In subgroup analysis, TcB in babies 28-33^6/7^ weeks of gestation (r = 0.944; p < 0.001) are nearly equally correlated with late preterm neonates (r = 0.950, p < 0.001). The bias difference between TcB and TSB in lower preterm groups and late preterm groups was 1.24 mg/dl and 1.1 mg/dl, respectively.

## Discussion

In the present study, TcB is excellently correlated (r = 0.94, p ≤ 0.001) with TSB in preterm neonates prior to phototherapy. The correlation coefficient between TcB and TSB among lower-gestational-age neonates (r = 0.944) is comparable to late-preterm neonates (r = 0.950). In the published literature available, this is the largest series of preterm neonates with regard to estimation of TcB by JM-105, advanced equipment for non-invasive bilirubin estimation. Already two systematic reviews and meta-analyses by Nagar et al. and Shabuj et al. depict the reliability of TcB devices (JM-103 and Bilicheck) in preterm infants [5,6]. The TcBmeters like JM-103 or Bilicheck reliably estimated bilirubin levels in preterm infants; however, the JM-103 device exhibited better precision than the Bili Check. The JM-105 used in this study is the next generation of JM-103, with advanced features in the basic functionality, i.e. measuring probe, hardware, and software used for measurements [7]. TCB measured by JM-105 is more reliable (r = 0.944) than JM-103 or Bilicheck (pooled correlation coefficient around 0.83 mentioned in the systematic reviews) [5,6].

Similar to previous studies by Mandal et al. [8], Pendse et al. [9], Pratesi et al. [10], and Kitsommart et al. [11], a significant correlation was observed here between TcB and TSB values. The correlation coefficient of TcB and TSB in this study is incongruent to Pendse et al.'s with a similar gestational age group in the Indian preterm neonatal population using JM-105. However, in contrast to our findings, the correlation coefficient is higher in the lower gestational age group compared to higher gestational age babies [9]. In a study by Kumar et al., a better correlation of TcB was noticed among lower gestational age compared to higher gestational age groups, but TcB was measured here by Bilitest-2000 [12]. Negar et al. saw the maximum correlation in 33-37 weeks of gestation and birth weight more than 2500 grams in Iranian preterm neonates using JH 20-1A for TcB measurement [13]. The accuracy of TcB in preterm neonates could be modified by gestational age, racial factors, sickness of the neonate, and types of equipment.

In the Bland-Altman plot of this study, the bias line indicated the mean difference of 1.16 mg/dl between TcB and TSB. The majority of the data points fell within ±1.96 times the SD of the difference between TSB and TcB values. This corroborates that there is a strong agreement present. The mean difference between TSB and TcB in preterm neonates before phototherapy is 0.64 mg/dl [95%: 0.13-1.14] in Posada et al. using JM-105 [14]. The slightly higher accuracy of Posada et al. could be secondary to the difference in the gestational age distribution and racial difference. Overall, TcB meter is handy, easy to carry out at the bedside, gives the result instantly, and decreases the frequency of painful pricking [15].

Limitation of the study

The study is monocentric with limited numbers of extremely-low-birth-weight neonates. It was done in clinically jaundiced preterm neonates before phototherapy rather than during phototherapy or after phototherapy to evaluate its accuracy during rebound hyperbilirubinemia.

## Conclusions

The TSB level estimated by the conventional method has a significant correlation with the value of bilirubin as measured by the transcutaneous bilirubinometer in preterm neonates. The use of a transcutaneous bilirubinometer will decrease the frequency of painful phlebotomy in preterm neonates and will also decrease parental anxiety. We recommend that transcutaneous bilirubinometer can be effectively used as a screening tool to predict bilirubin levels even in premature babies and can be used to dictate management guidelines. Further studies with a larger cohort of babies, and estimating TcB after phototherapy and correlating with TSB will further help in strengthening transcutaneous bilirubinometer as a diagnostic device. As per our study, the values of TcB correlated very well with TSB measured in the laboratory by standard methods and should be used in screening and deciding therapeutic interventions in premature neonates as well.
